# Newsy Letter from San Antonio

**Published:** 1889-07

**Authors:** D. Berry

**Affiliations:** Sec. W. T. Med. Association


### CORRESPONDENCE.

### NEWSY LETTER FROM SAN ANTONIO.

Editor Daniel's Texas Medical Journal:

Of late I have been surprised to see the few contributions to the columns of your JOURNAL from the profession of this city. Northern and Eastern Texas is always well represented, but it is only occassionally that you see a contribution from this section of the State. This should not be so, for we have many men in West Texas who are clever writers and able thinkers, and whose experience, if jotted down from time to time and published in some journal, would prove of much practical value to the profession at large. The general practitioner is far more interested in the short practical article, giving facts gathered from every-day experience, rather than the long, dry scientific discourses written on some subject which is perhaps totally devoid of interest to the average practitioner.

The profession of San Antonio have been much pleased with the many flattering comments which have appeared in the medical press, from time to time, regarding the entertainment of the Texas State Medical Association at their last meeting in this city. All was done that money and hard work could do to make the meeting a success, and it gratifies us not a little to see that our efforts have been so much appreciated. Our own ideas regarding banquets are perfectly in accord with the acticle in the June number of the JOURNAL, from the pen of Ex-Vice President O. Eastland. I am sure that the better interests of the Association can be subserved without the annual banquet. About $2500 was raised by the profession of San Antonio for the entertainment of the State Association, and of that amount over $1200 was spent in a single banquet; and is there a single man who sat down to *that* banquet but who is satisfied that the money could have been far better spent? So even now permit me to add my protest against a banquet at our next annual meeting in Fort Worth. Let the finger of that committee of arrangements "point to the snow capped heights of Pike's Peak rather than to the banquet hall," and Fort Worth will have such an attendance as has never been recorded in the annals of the State society.

The West Texas Medical Society is doing good work; new members are being added at almost every meeting, and the best feeling prevails. At the last regular meeting of the West Texas Medical Association, held June 30, the following circular, which explains itself, was decided upon:

"DEAR DOCTOR—When the West Texas Medical Association was organized in 1876, the object of that organization was for a Medical Association for Western Texas, as its name implies, and it was hoped that every regular physician in good standing west of the Colorado river would be enrolled in its membership, but we are sorry to say that but few physicians outside of this city have joined us. In the past few years the North Texas Medical Association, the Central Texas Medical Association and the Austin District Medical Association have organized (subordinate to the Texas State Medical Association), and are all doing good work, and it is time that every physician in West Texas should be up and doing. We should organize and move abreast with these societies in their laudable efforts for the advancement of the profession. It is proposed that the West Texas Medical Association take a new departure, and it was determined by resolution, at the last regular meeting, to have quarterly meetings for discussions of medical, surgical and scientific subjects, and the undersigned were appointed a committee to communicate with the regular physicians of Western Texas, and urge them to meet us on Wednesday, July 31, at 10 a. m., for the purpose of organizing for the work that is before us. Doctor, let us beg you, in the interest of the profession of the whole State, to come and give us your valuable assistance in this enterprise, and if yon have a neighbor whose name we have not gotten, extend the invitation to him."

Respectfully,

GEO. CUPPLES, M. D.,
T. R. CHEW, M. D.,
C. E. R. KING, M. D.,
D. BERRY, M. D., Sec.,
Committee.

About 250 of these circulars have been sent out to physicians in the western section of the State, and a large attendance is expected. In the last few days two medical examining boards have been appointed, in the 32nd and 45th judicial districts; all the appointees being San Antonio men.

Judge Noonan. of the 32nd judicial district, appointed Drs. F. Herff, T. R. Chew, E. Bennett, G. G. Watts, E. Hertzberg, P. Ornelus and S. T. Lowry.

Judge W. W. King, of the newly created 45th judicial district, appointed Drs. Geo. Cupples, P. W. Johns, J. Braunnagel, R. Menger, L. L. Shropshire, Jos. Jones, (homeopath) and George Clifford, (homeopath).

Surely, when the enterprising irregular and the long-haired fakir become aware of the fact that they will have to face such an array of medical talent, they will give San Antonio a wide berth. More anon.

Respectfullly,

D. BERRY, M. D.,
Sec. W. T. Med. Association.

